# Overview of Clinical Relevance of Antibodies Against Oxidized Low-Density Lipoprotein (oLAb) Within Three Decades by ELISA Technology

**DOI:** 10.3390/antiox13121560

**Published:** 2024-12-19

**Authors:** Willibald Wonisch, Franz Tatzber, Meinrad Lindschinger, Andreas Falk, Ulrike Resch, Sabrina Mörkl, Neven Zarkovic, Gerhard Cvirn

**Affiliations:** 1 Otto Loewi Research Center, Division of Medicinal Chemistry, Medical University of Graz, 8010 Graz, Styria, Austria; gerhard.cvirn@medunigraz.at; 2 Omnignostica Ltd., 3421 Höflein an der Donau, Lower Austria, Austria; 3 Institute of Nutritional and Metabolic Diseases, Outpatient Clinic Laßnitzhöhe, 8301 Laßnitzhöhe, Styria, Austria; meinrad@lindschinger.at (M.L.); andreas.falk@bnn.at (A.F.); 4 BioNanoNet Forschungsgesellschaft mbH (BNN), 8010 Graz, Styria, Austria; 5 Department of Vascular Biology and Thrombosis Research, Medical University of Vienna, 1090 Vienna, Vienna, Austria; ulrike.resch@meduniwien.ac.at; 6 Department of Medical Psychology, Psychosomatics and Psychotherapy, Medical University of Graz, 8036 Graz, Styria, Austria; sabrina.moerkl@medunigraz.at; 7 Laboratory for Oxidative Stress, Division of Molecular Medicine, Rudjer Boskovic Institute, HR-10000 Zagreb, Croatia; zarkovic@irb.hr

**Keywords:** antibody against oxidized LDL, ELISA, antioxidants, oxidative stress, lipid peroxidation

## Abstract

One of the most prominent actions of oxidative stress is the attack of free radicals on poylyunsaturated fatty acids (PUFAs), initiating a chain reaction to modify these PUFAs and generate oxidized modifications on all biomolecules. In the last quarter of the 20th century, intensive research was carried out to identify antibodies against such modifications. In the mid-1990s, the first enzyme-linked immunosorbent assay (ELISA) was introduced to the market, significantly accelerating research activities and knowledge gain. During this pioneering period, the main focus was on cardiovascular diseases, cancer, diabetes, and other diseases associated with oxidative stress. Subsequently, a standard range of these antibodies against oxidized LDL (oLAb) was determined in the population. Furthermore, the impact of exhaustive physical activity and diet on oLAb titers, and the correlation between newborns and mothers after delivery, as well as nutritional intake in newborns, were evaluated. Subsequently, the harmful effects of smoking and many other areas regarding oLAb titer were published, resulting in novel approaches for prognostic and therapeutic options, in particular through studies with antioxidants, which were able to influence oLAb significantly. This review presents an overview of the research activities obtained with this ELISA over the past three decades.

## 1. Introduction

Oxidative stress (OS) is defined as an imbalance in the redox cycle characterized by an excess of oxidants in relation to antioxidants, which disturbs the sensibly balanced equilibrium between these redox categories [1]. For many decades, numerous researchers have provided evidence that oxidative stress can be either caused by the excessive consumption of pro-oxidants, the excessive endogenous production of reactive oxygen species, or the lack of antioxidants [2,3]. One of the most prominent consequences of OS is the attack of free radicals on polyunsaturated fatty acids (PUFAs), initiating a chain reaction that decomposes PUFAs to generate highly reactive aldehydes such as malondialdehyde (MDA) and 4-hydroxynonenal (4-HNE), colloquially termed “rancid lipids” in the course of the so-called “lipid peroxidation” (LPO) cascade, which was extensively studied in the 1990s on the PUFA and cholesterol-rich lipoprotein particle Low-Density Lipoprotein (LDL), among others, in the lab of Hermann Esterbauer, who passed away in 1997 [4,5,6]. Cardio-vascular diseases and malignancies are the top two causes of death in developed countries. Both disorders are closely related to OS and LPO, which are partly responsible for initiating and exacerbating pathological processes, as postulated already by Brown and Goldstein in 1983 and demonstrated in numerous studies [7,8,9,10] (see Table 1 for details).

**Figure 1:**
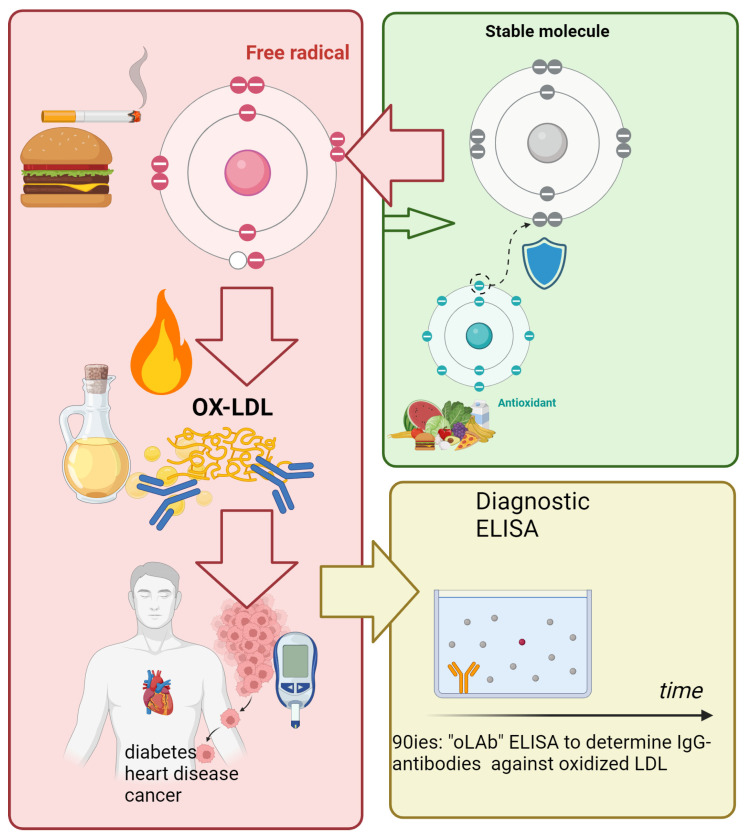
Reactive oxygen species (free radicals) attack PUFAs (among others) to generate lipid peroxidation products that modify proteins, making them immunogenic, which triggers the immune system to create antibodies against oxidized LDL (oLAb). The levels of oLAb can be measured using ELISA. Figure created with biorender.com.

The group of Steinberg, Witzthum, and Partharsarati [11,12] were the first to measure immunoglobulins reacting with aldehyde-modified proteins such as oxidatively modified LDL (oxLDL) in animal studies and to demonstrate that LPO occurs in vivo. Enzyme-linked immunosorbent assays (ELISA) coated with oxLDL were used to detect IgG antibodies in humans and several carnivorous and omnivorous animals [13,14] (see Figure 1).

Life-threatening conditions are associated with dramatic decreased titres of antibodies against oxidized-low-density lipoprotein (oLAb) in organisms, as could be shown in starved animals or humans suffering from septicemia, as well as significant decreases in non-surviving SARS-CoV-2 patients or during acute myocardial infarction. Physiologic antibodies, including oxLDL antibodies, are present in all mammalian species and are enormously important for the immune system to maintain homeostasis. These physiologic autoantibodies are produced in healthy individuals and perform protective and regulatory functions as a defence against diseases or for self-treatment after the onset of a disease. For instance, ageing erythrocytes show that immunoglobulin G (IgG) autoantibodies bind to senescent antigens on aged cells. Oxidation generates these neoantigens, which the immune system recognizes. As soon as the IgG is bound, the removal of the senescent cells is accelerated and carried out by macrophages [15]. Oxidized LDL expresses a large and, to date, unknown number of epitopes that induce the production of antibodies against these products [16]. Since the mid-1990s, the Biomedica company has been a pioneer in producing the “oLAb” ELISA to determine IgG-antibodies against oxidized LDL based on the work of Tatzber and Esterbauer 1995 [17]. For comparability reasons, this review comprises exclusively studies performed with this commercial standardized method as works using custom-made assays are reviewed elsewhere [18]. With the help of this methodology, numerous studies have been conducted in recent decades, and findings regarding the immunological level of oxidative stress in various diseases have been made, which have benefited humankind and medicine [19,20,21]. In this context, Winklhofer-Roob et al. [22] mentioned the oLAb as fingerprints of an immune response to oxidized LDL. Another review stated that the assessment of oxLDL antibodies may more reliably reflect the level of oxidative stress than oxLDL [23].

Future aspects should be primarily concerned with the early diagnostic detection of excessive oxidative stress and initiating therapeutic intervention in preventative health care. Finally, an outlook on future aspects and possible applications of various antibodies against oxidized LDL, e.g., the application of human monoclonal antibodies against oxidatively modified lipoproteins may open new therapeutic concepts in the fight against life-threatening diseases like malignancies, septicemia, and cardiovascular diseases.

## 2. Antibody Titer Against Copper-Oxidized LDL in the General Population

At the beginning of antibody research against oxidized low-density lipoprotein (oxLDL) in the early 1990s, evidence accumulated that atherosclerotic disease is coupled with an elevated oxLDL antibody titer, and thus estimated as a health hazard [24]. Pincemail et al. [25] were the first who assessed and published the normal range for the antibody titer against oxidized LDL (oLAb) in the general population (*n* = 123) in an age range between 21 and 64 years. They determined a mean value of 468.37 ± 318.55 mU/mL with a normal range between 195 to 600 mU/mL. This standard range was substantiated in a large-scale study [26] with a random sample of 2793 participants (52.4% female) and an age range between 25–74 years. The median oLAb-titer was 300 mU/mL (5th–95th percentile: 74.4–1681) for males and 363 mU/mL (5th–95th percentile: 80.4–1810) for females.

The antibody titer in pregnant women (*n* = 40, age 27 ± 4.1 years) is not significantly different between the first and second trimesters (561.5 ± 424 vs. 581 ± 434 mU/mL) [19]. Nevertheless, in a comparative study of normotensive pregnant women (*n* = 49) with 26 women affected by pregnancy-induced hypertension, a significantly decreased oLAb titer associated with hypertension (348 ± 388 mU/mL, vs. 579 ± 400 mU/mL, *p* < 0.01) was observed. This is of interest for neonates, as their oLAb titer is similar to their mother’s post-partum titer due to transplacental transport. Delivery is associated with oxidative stress for both mothers and newborns, as indicated by significantly increased levels of the LPO-product MDA versus controls, and even higher concentrations in newborns accompanied by a decreased abundance of the antioxidant enzyme superoxide dismutase (SOD). Consequently, the oLAb titer doubles within the first three months in newborns, while antioxidative glutathione (GSH) levels decrease significantly. Furthermore, lipid parameters were significantly increased after three months [27]. This working group of Steinerova et al. proved that the antibody titer depends on nutrition in newborns after three months, although there is an initial oLAb correlation with their mothers (r = 0.79; *p* < 0.001). In detail, breast-fed babies showed an average oLAb titer of less than 100 mU/mL, while formula-fed babies showed an oLAb titer of about 4000 mU/mL. This discrepancy could be due to a reaction to foreign proteins from cow’s milk or modified molecules, especially since increased DNA damage was identified in formula-fed infants associated with oxidized pyrimidines. They identified a bovine milk protein, i.e., beta-casein A^1^, as being atherogenic, thus, almost exclusively male formula-fed infants produced high levels of oLAb in consequence [28,29]. However, as an Italian research group has shown, the diet-dependent influence on the oLAb titer is not limited to newborns. They conducted a prospective population-based longitudinal investigation, including 101 males and 164 females, of pharmacologically untreated non-smoker subjects, who were evaluated for factors that might affect their oLAb titers. Although they did not observe significant effects of the dietary approach, which did not influence the oLAb titer in a clinically relevant manner, they observed a significant predictor of the log-ox-LDL antibody level in a multivariate analysis for age (HR −0.19; 95% CI −0.115–−0.104, *p* = 0.001; adjusted r^2^ = 0.581). In males, the log-ox-LDL antibody level was only predicted by the current dietary animal lipids content (HR −0.12; 95% CI −0.127–−0.109, *p* < 0.001; adjusted r^2^ = 0.128) in comparison to females, where age (HR −0.11, 95% CI −0.119–−0.107, *p* < 0.001), soluble fibers (HR 0.11, 95% CI 0.104–0.110, *p* < 0.001), and alcohol intake (HR 0.18, 95% CI 0.102–0.114, *p* < 0.013, adjusted r^2^ = 0.182) were the best predictors of oLAb levels [30]. Antibodies against oxidized LDL were not significantly different between alcoholic patients with slightly affected liver function but without any disease (*n* = 35, age 42 ± 9 years) compared to healthy blood donors (*n* = 60, age 40 ± 8 years) [31]. The use of oral contraception in 40 to 48-year-old females was associated with a significant pro-oxidative effect through total increased peroxides compared to non-contraception users or intrauterine device users. Elevated copper concentrations decreased β-carotene and **γ**-tocopherol without affecting the oLAb titer. The strong correlation between copper and total peroxide increase indicates lipid peroxidation enhancement in females using oral contraception. These authors conclude with the comment to monitor these parameters and suggest antioxidant supplementation considering the potential risk for venous thromboembolism and cardiovascular disorders [32]. In the Wandsworth Heart and Stroke Study population, different healthy ethnic groups, i.e., 250 Whites (113 females), 169 African origin (91 females), and 196 South Asian (92 females) were determined for soluble serum oLAb levels [33]. The oLAb titer was significantly increased in subjects of African origin and South Asian origin. The titer was negatively associated with triglycerides and positively with s-VCAM-1, especially in female South Asian individuals, with a decrease in Vitamin C level and an increase in plasma homocysteine concentrations, which indicates a condition of increased risk for coronary heart disease (CHD) and cardiovascular disease (CVD).

The ethnic influence on the oLAb titer is also evident from a study of 158 women and 158 men in Japan [34], with relatively low oLAb mean values (female 209 (152.6–312.5); male 170.7 (130.9–301.2)) compared to the standard European normal range [25]. A large-scale cross-sectional study from Poland, including 3154 individuals aged 65 and older, showed that advancing age was significantly associated with decreased paraoxonase 1 (PON1) activity and increased oLAb. The age-dependent diminished activity of PON1 was related to inflammation and the generation of oLAb as a counterbalance to elevated oxidative stress. In detail, the authors observed a significantly lower oLAb titer in males than females, and an inverse relation to triglycerides. They concluded that both PON1 and oLAb act independently and synergistically against the amount of oxLDL as PON1 activity decreases with age. Thus, oLAb becomes more critical in eliminating oxLDL with extraordinarily high titers in the oldest group [35].

### 2.1. oLAb and the Innate Immune System

**Figure 2:**
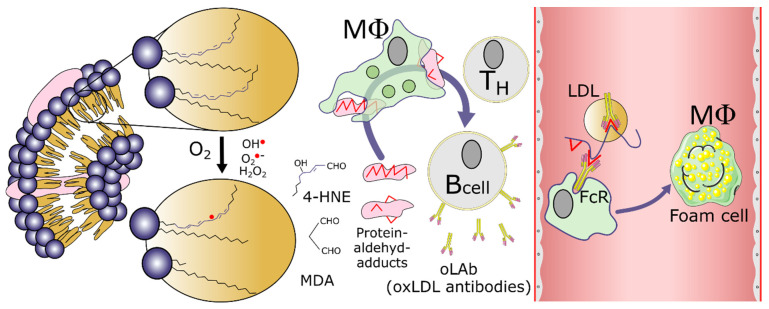
Left part: oxidative stress gives rise to free radicals, which initiate and promote the decomposition of polyunsaturated fatty acids (PUFA) in the cell membrane’s or LDL phospholipid Sn2-position to produce highly reactive aldehydes such as MDA and 4-HNE, which react with proteins in close vicinity such as ApoB on LDL. Damaged proteins are phagocytosed by macrophages (MΦ), which process them in lysosomes into smaller peptides, which are then presented as antigens to immune cells, and B-cells produce IgG antibodies recognizing oxidatively modified proteins, such as ox-LDL. Right part: in the circulation, oxLDL is recognized by oLAb and cleared from circulation via FcR (Fc-Receptor) on macrophages. The continuous uptake of oxLDL by macrophages gives rise to foam cell formation.

The oxidation of LDL through ROS, including hydroxyl radical (OH^•^), superoxide anion (O_2_^•−^), or hydrogen peroxide (H_2_O_2_) leads to the formation of modified phospholipids with a surface structure similar to that of damaged cells, which are recognized by antibodies against oxidized LDL and scavenger receptors, which also play an essential role in the recognition and clearance of bacteria and apoptotic cells [11]. Oxidatively modified LDL binds to the scavenger receptor on macrophages, which are converted to foam cells by uncontrolled intake as indicated in the scheme in Figure 2.

In this respect, it should be noted that pneumococcal vaccination is associated with an increase in oLAb, indicating a mechanistic link between vaccination and a protective effect on cardiovascular disease. After multivariate adjustment for potential confounders, this association remained significant (*p* = 0.04). This seems particularly important for older people, and pneumococcal vaccination is now recommended for these subjects as their immune systems weaken with age, and they are at an increased risk of vascular diseases [36].

### 2.2. Smokers

Heavy smokers (>20 cigarettes/day) showed a significant increase in the oxidative stress DNA marker 8-hydroxy-deoxyguanosine (8-OHdG), associated with a significant decrease in α-carotene, β-carotene, β-cryptoxanthin, and zeaxanthin. In males, a significant negative correlation was shown between oLAb and β-carotene (r = 0.33, *p* < 0.01) with the number of cigarettes per day. Thus, the authors recommended 8-OHdG, oLAb, and carotenoids as valuable biomarkers to evaluate the oxidative conditions caused by smoking [37]. In an ethnic study [33], it was found that, in smokers, the oLAb titer was significantly decreased (384 U/L, 95% CI 316–468 U/L) compared to in non-smokers (430 U/L, 95% CI 471–596; *p* < 0.009). In stenotic patients, an increased oLAb titer was observed in smokers (472.2 ± 65.9 mU/mL) compared to in non-smokers (275.6 ± 33.5 mU/mL). Increased oLAb titers were significantly pronounced in unstable angina (*p* < 0.005) [38].

### 2.3. Body Mass Index (BMI)

Body mass index is associated with increased insulin resistance and hyperglycemia in elderly men, especially in “young-old men (62–74 years)” with a significantly increased BMI and decreased oLAb compared to “old-old men (75–83 years)” [39]. Malondialdehyde and oLAb were not significantly different between young and elderly, except for a trend of lower oLAb titers in the elderly and a direct relation between MDA and oLAb, independent of age. Serum antioxidant status was also similar between age groups, with an inverse relationship between oLAb, Vitamin C, and Vitamin E [40]. The inverse relationship between BMI and oLAb was confirmed in a cohort of 2190 subjects (1283 females), showing a significant oLAb decrease with the lowest titer at BMI > 30. This correlated with the highest cholesterol, LDL, uric acid, and peroxide concentrations, while bilirubin and HDL were lowest in this BMI group. A linear increase in the antioxidant status (TAS) occurred up to a BMI of 34.9, presumably due to the steady increase in uric acid, an excellent antioxidant correlating significantly with the TAS. In those subjects with a BMI of 35 and above, despite a further increase in uric acid, there was a decrease in TAS coupled with a doubling of peroxides [41].

In a study with anorexia nervosa (AN) patients, the lowest oLAb titres were measured in the lower subcutaneous adipose tissue (SAT) group (BMI = 14.5 ± 1.1) [42]. Although the antioxidative and oxidative stress biomarkers were below reference values in the higher SAT group (BMI = 16.1 ± 0.9), these parameters were still better than those in the lower SAT group. This is crucial information, especially since a previous study in subjects generally defined by a body mass index (BMI) < 18.9 showed no significant differences in oLAb levels compared to normal weight subjects [41]. Therefore, conducting a detailed oxidative stress analysis for AN-patients is essential and reasonable, as this group with lower SAT is associated to an extraordinary degree with increased oxidative stress, which also impacts the therapeutic outcome and long-term recovery.

### 2.4. Competitive Sports

Exhaustive exercise is associated with increased oxygen consumption and metabolism. Thus, reactive oxygen species (ROS) become elevated during training and competition, which induces the generation of oLAb to protect against oxLDL damage and further consequences against atherosclerotic modifications. Half of soccer and basketball players indicate very high titers of oLAb—up to 6000 mU/mL. This was accompanied by a trend towards lower vitamin C levels in athletes with high oLAb titres (8.49 ± 3.14 µg/mL vs. 10.39 ± 2.55 µg/mL), apart from the fact that it was not significant [25]. In another study, the majority of soccer players (*n* = 7) were indicated with a mean oLAb titer of 1102.8 ± 45.8 mU/mL and four players with 31.5 ± 12.6 mU/mL, although antioxidant levels were in the normal range and did not differ between groups [43]. Nansseu et al. [44] reported a significant increase in the oLAb titer during the competition season, as indicated at three time points (median oLAb titer 653 mU/mL March; 777 mU/mL May; 1037 mU/mL July; *p* = 0.006) in eighteen professional soccer players. They further observed a concomitant decrease in uric acid and total antioxidant capacity.

In professional American football players, likewise in half of the athletes, a significant increase in total peroxides and oLAb was shown in the middle of the competition season, which levelled off at the end. The oxidative stress was neither predictable from baseline values nor on the status of individual antioxidants [3]. The oxidative stress in the course of a competition season (July–January) in the Austrian Men’s Alpine Ski Team showed a high load in December, whereby the basic oLAb titer (1036 ± 328 mU/mL July) was decreased to 439 ± 150 mU/mL, which indicates a significant consumption of these antibodies. The athletes recovered to near pre-season levels during the days off over Christmas and New Year. The oxidative stress parameters also correlated with the athletes’ performance, with the successful athletes having only about 50 per cent of the peroxide concentrations and an oLAb titer at least twice as high as the low-performing skiers. In addition, the latter were also susceptible to infections in December [45]. In this context, it should be noted that the antioxidant concentrations were, on average, all within the normal range (α-tocopherol, γ-tocopherol, ascorbate serum concentrations) or, in the case of β-carotene, even twice as high as the reference values. The oLAb titer of active cyclists decreased significantly compared to that of the control group, and the titer of ex-cyclists was between these two groups, although not significantly different from that of the control group. The training effect remained for the improved antioxidant status for one year, while the effect on oLAb was lost as soon as one became sedentary. Interestingly, there was no correlation between training hours and the oLAb-titer [46].

Regarding non-exhausting conditions in a group of 41 postmenopausal female members (age 59–71 years) who completed a training program over eight weeks (three times a week) on an ergometer, the oLAb proved to be a robust parameter that remained constant under these health-promoting conditions, primarily as the anthropometric parameters such as BMI or body weight also remained unchanged. However, the positive effect of physical activity had an impact on glucose levels, LDL-cholesterol, and antioxidant capacity [47]. The same study group performed an aquatic training programme, i.e., one hour twice a week for three months with 12 obese women (aged 44–61 years). The programme improved glucose tolerance, decreased the HOMA (IR) index, and reduced the total LDL cholesterol levels. However, no changes were observed in adiponectin levels, body mass, or oLAb [48].

### 2.5. Hyperlipidemia

A low-grade inflammatory state, metabolic disturbances in the lipid profile, and insulin resistance occur in Familial Combined Hyperlipidemia (FCH). It is associated with an increased oLAb titer, even though the oLAb-titer of the control group was in the lowest section of the normal range [49]. In a small number (five males and one female in the range of 41–60 years) of heterozygous familial hypercholesterolemia subjects, a median oLAb titer of 269 mU/mL was observed before and 315 mU/mL after LDL apheresis, which was not significantly different. This form of treatment did not change the level of 8-iso-PGF2***α*** but increased the lag time and decreased the concentration of TBARS [50].

### 2.6. Diabetes Mellitus

In the case of insulin-dependent diabetes mellitus patients, the severity of the disease was inversely related to the oLAb titer, indicating the requirement of these antibodies to react with the antigen, as oxLDL immune complexes were identified in those patients with longer diabetes duration and higher actual and mean HbA1c levels [51]. Childhood type 1 diabetes mellitus (T1DM) is associated with significantly higher oLAb titers than controls, which indicates an inverse correlation between oLAb and HbA1c levels. This is an immunological defense against an excess of oxidative stress. In detail, those subjects with reasonably good metabolic control (*n* = 21), i.e., HbA1c levels less than or equal to 9%, have shown a marked increase in oLAb (488 mU/mL). In contrast, the poor metabolic control (*n* = 15) showed a markedly decreased antibody titer (183 mU/mL), most probably due to the worse redox balance by forming more antigen–antibody complexes reflected by a lower titer of free antibodies [20]. In young type 1 diabetic patients, the oLAb titer decreased with age and the duration of diabetes, which fits atherosclerotic processes as oLAb is an immunologic expression of lipid peroxidation. In this respect, the inverse correlation with vitamin E can also be classified, with the lipid-soluble antioxidant representing a significant factor in protecting against the in vivo oxidation of LDL. On the other hand, there were no significant differences in oLAb titers in these young diabetics, who were generally under reasonable diabetic control, regardless of whether they had no complications, subclinical retinopathy, neuropathy, or nephropathy [52].

### 2.7. Antioxidants

The benefits of polyphenols in olive oil were established in a clinical trial [53] as they promote an increase in oLAb titers, with the most pronounced effect in subjects with high oxidized low-density lipoprotein (ox-LDL) concentrations. The oLAb titer increased with the polyphenol content in a dose-dependent manner and was inversely associated with oxLDL. This was substantiated by the correlation of the oLAb titer with the amounts of polyphenols bound to LDL cholesterol. Another comparative study between Iranians and Austrians [54] showed that Iranians indicated a higher oLAb titer and lower cholesterol, LDL, and HDL levels, but increased levels of lycopene, canthaxanthin, and lutein. An intriguing perception in this study was the significantly increased oLAb titer (*p* < 0.01) accompanied by an increased resistance of LDL (lag phase; *p* < 0.005) with a concomitant decrease in MDA levels after a combined daily intake of beta-carotene (30 mg) and alpha-tocopherol (400 IU) over ten weeks. Based on these studies, the intake of fat-soluble antioxidants appears to increase the oLAb titer. On the other hand, pomegranate juice (200 mL per day for six weeks), which is abundant in polyphenols, prevented oxidative stress in type 2 diabetic patients, reduced the oLAb titer, and increased the total antioxidant capacity and PON1 activity [55]. In a cross-sectional study with 551 community-dwelling older adults between 65 and 94 years old from Dicomano, Italy, it was shown that wine consumption, through its antioxidant properties, affects oLAb titers in older populations [56]. Oxidative stress was significantly reduced during a 24-week therapy by Rosuvastatin, including an immunomodulatory effect. This statin, which has antioxidative effects, attenuates oxidative stress through a decrease in total peroxides and antibodies against oxidized LDL, in addition to lowering cholesterol, thus supporting the prevention of atherosclerotic diseases [57]. Young patients with bipolar disorder exhibited an inverse association between vitamin D and the oLAb titer (r = −0.34; *p* < 0.05) [58].

## 3. oLAb and Cardio-Vascular Diseases

### 3.1. Systemic Sclerosis

In patients suffering from Systemic Sclerosis (SSc), the oLAb titer increased predominantly in the early phase in both the limited and diffuse SSc (428.9 ± 417.1 mU/mL), where the immune response to lipid peroxidation products is much stronger than in the advanced phase (302.7 ± 89.9 mU/mL) of the disease. Nevertheless, the pathophysiology of the limited form is predominant and associated with extended reperfusion injury compared to the diffuse SSc, where the episodes are significantly fewer due to the loss of capillaries [59].

#### 3.1.1. Peripheral Arterial Disease (PAD)

In twenty-one diagnosed patients by peripheric angiography suffering from peripheral atherosclerotic disease (PAD), the oLAb titer was increased (357.44 ± 177.71 mU/mL) compared to the control group (266.35 ± 113.19 mU/mL; *p* < 0.05). Moreover, in patients with severe Fontaine claudication (IIb), the oLAb titers were higher (403.03 ± 223.67 mU/mL) compared to patients with mild Fontaine claudication (IIa) (316.00 ± 119.02 mU/mL). As a result of increased oxidized LDL in these patients, the increased oLAb titer is of great importance due to its physiological function of removing oxLDL from the circulation and the artery wall [60]. Vascular walls are damaged during percutaneous transluminal angioplasty (PTA), which is finally associated with ischemia/reperfusion injury. This technique is primarily performed in patients with Fontaine stage IIb and IV. The latter group is characterized by significantly increased peroxide concentrations, while revascularization is associated with a significant decrease in oLAb titers, which is why this antibody could be important as a revascularization marker [61].

#### 3.1.2. Coronary Balloon Angioplasty

The prognostic value for oLAb was investigated in patients undergoing primary coronary balloon angioplasty for acute ST-elevation myocardial infarction (STEMI). Restenosis was observed in approximately one third of patients. In this group, the oLAb titers were significantly decreased before angioplasty, while oxidized LDL did not differ between groups. Therefore, a sufficient titer of oLAb is a prerequisite for the clearance of ox-LDL from circulation to avoid restenosis after primary angioplasty [8].

#### 3.1.3. Cardio-Vascular Disease (CVD) and Intima Media Thickness (IMT)

In healthy subjects, a significant inverse association between oLAb titers and the intima-media thickness of the carotid arteries, even after multiple regression analysis, indicated the immune response as a safeguard against oxidized LDL and its detrimental health implications, such as at an early stage of atherosclerosis [62].

In cardiovascular disease (CVD) patients, an increased ox-LDL was associated with decreased oLAb titers. Intima-media thickness (IMT) values were higher in uremic patients versus controls, and in CVD patients compared to patients without CVD, which correlated positively with the ox-LDL/oLAb ratio in comparison to an inverse association to oLAb. The authors suggested the ox-LDL/oLAb ratio as a new predictor of IMT, with an important role in distinguishing between patients with and without cardiovascular complications [63]. The inverse association between oxLDL and oLAb was also reported in male subjects with stable coronary heart disease (CHD). A significant increase in ox-LDL was associated with the consumption of oLAb and an increase in Superoxide-Dismutase (SOD) and glutathione-peroxidase activity (GSH-Px) [64]. In a clinically healthy population, plasma ox-LDL concentrations were also negatively correlated with oLAb regarding the intima-media thickness of the common carotid arteries. Those subjects with high oLAb and low ox-LDL had the most negligible intima-media thickness of the common carotid arteries [65]. The concept that oLAb, i.e., the immunity against oxidized low-density lipoprotein, plays an anti-atherogenic role was supported by the significant inverse relation between oLAb titer and intima-media thickness of femoral arteries (FA-IMT), and the inverse trend between oLAb and intima-media thickness of carotid arteries (CA-IMT) in end-stage renal disease patients [66]. The greyscale median of the intima-media complex (IM-GSM), a tool for atherosclerosis detection alongside the intima-media thickness, was associated with several inflammatory markers and oxidative stress biomarkers, among others with an inverse relationship to oLAb in an elderly population with 1016 participants from the community of Uppsala, Sweden [67]. In CAD patients with no vessel disease, the oLAb titer was significantly increased compared to controls. This finding indicates an advanced generation of these antibodies as a countermeasure upon an increased incidence of antigen. In advanced stages of the disease, i.e., with single-, double-, or triple-vessel disease, these antibodies are consumed, which could be determined by decreased titers [68].

#### 3.1.4. Angina Pectoris

A study of patients diagnosed with angina pectoris showed an increased oLAb titer of 27% compared to the control group [69].

#### 3.1.5. Acute Myocardial Infarction (AMI)

The dynamic of oLAb titers is probably best pictured in acute pathological settings such as AMI, and it is thus not surprising that seemingly contradictory results were reported by several research groups. Stable acute myocardial infarction (AMI) patients without classical risk factors were identified with very low oLAb titers, significantly differing from population controls indicating an oLAb cut-off value for AMI risk at 165 U/L, applying a bootstrap method with 1000 replications. Patients with an oLAb titer of 165 U/L or less had a higher risk of AMI with a crude odds ratio (OR) of 6.15 (95% confidence interval (CI): 2.24–16.9) and after adjusting by the Framingham-risk-adapted score and ox-LDL, the natural logarithmic level of oLAb maintained the independent association (OR of 0.43, 95% CI: 0.23–0.79). On the other hand, the ox-LDL level between controls and cases did not differ [70]. The working group of Gruzdeva et al. reported that the oLAb titer in myocardial infarction patients (MI) was significantly increased compared to controls. In addition, complicated MI (Killip class II-IV) had higher oLAb titers on day one compared to non-complicated MI (Killip class I), which stayed high until day twelve [71]. The same group performed a study including 400 patients with myocardial infarction who were assigned to ST-elevation, which were divided into three groups, i.e., (A) one vessel ≧ 75% stenosis, (B) two vessels ≧ 75% stenosis, and (C) three or more coronary arteries with ≧ 75% stenosis and blood samples were drawn at the first and 12th days. In addition to increased lipid values such as free fatty acids, total cholesterol, LDL, triglycerides, peroxides, and oxidized LDL, significantly increased antibody titers against oxLDL were also determined compared to the control group. In group A, the titer was increased by 37% (*p* < 0.01), in group B by 69% (*p* < 0.005), and in group C by 134% (*p* < 0.004) on the first day compared to the control. The antibody titer also increased with the number of affected arteries, i.e., by 17% in group B and 70% in group C, compared to group A [72]. The authors concluded that FFA or antibodies against oxidized LDL would be the most informative indices for the severity of atherosclerotic lesions, which are elevated in multivessel disease during hospitalization.

On the contrary, the study by Nikolic-Heitzler et al. [73] confirmed the results of Schumacher et al. [13] regarding the initial consumption of oLAb after percutaneous coronary intervention due to acute myocardial infarction: a significant decrease after three days and a significant increase after another four days. In this respect, the measurement time points appear to be decisive since Schumacher et al. [13] monitored a period from admission to 48 h afterwards, and Nikolic-Heitzler et al. [73] from admission via 2 and 4 h as well as after 3 and 7 days, in contrast to Gruzdeva et al. [71] who analyzed only at two time points, i.e., after one and twelve days. An antibody titer increase was already reported after seven days [73].

#### 3.1.6. Stroke

**Figure 3:**
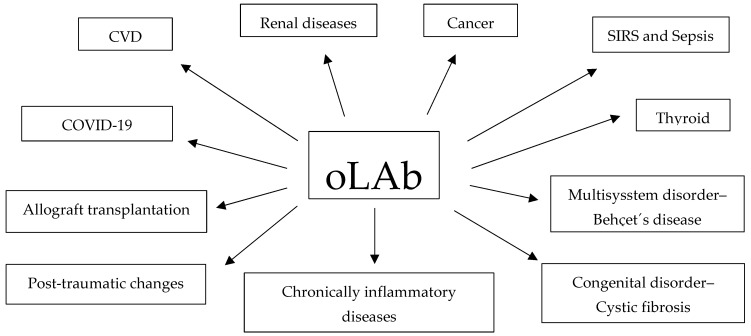
Scheme of different diseases and surgical interventions in which oLAb was determined.

The antibody-titer against oxidized LDL was significantly decreased in stroke patients (246.9 ± 38.2 mU/mL) compared to controls (505.8 ± 103.4 mU/mL), and was especially pronounced in hypertensive patients (199.5 ± 41.8 mU/mL). Cerebral ischaemia is associated with increased oxidative stress, and the decreased oLAb titer can be explained by binding to oxidatively modified structures [74]. Figure 3 provides an overview of the use of oLAb ELISA in various diseases and surgical interventions.

## 4. Renal Disease and oLAb

In renal disease patients, oLAb titers were both increased in patients after kidney transplantation and more pronounced in hemodialysis patients, presumably due to the atherogenic lipids and lipoprotein ratios [75]. In hemodialysis patients, superior to patients with cardiovascular disease, an increase of the vascular endothelial growth factor was associated and positively correlated with the Cu/Zn superoxide dismutase level (*p* < 0.01) and the oLAb-titer (*p* < 0.05). This indicates a local tissue production of ROS and VEGF following the hypothesis that the global activation of macrophages and smooth muscle cells, which synthesize and secrete these molecules, are responsible for their accumulation in plaques. This was supported by a multivariate analysis showing that Cu/Zn SOD and oLAb are strong independent variables linked to VEGF [76]. Predialysis patients with carotid plaques had significantly increased oLAb titers compared to those without plaques, as confirmed by Rubba et al. [77], who found an independent correlation between the plaques in the common carotid artery and the oLAb, but not in the bifurcation. An inverse correlation between oLAb and vitamin E indicates the protective effect of tocopherol against the oxidation of LDL, with malnourished chronic renal failure patients showing significantly lower vitamin E levels [78].

In patients with chronic renal failure, elevated VEGF levels were reported in association with an increase in inflammation, total peroxides, and Cu/Zn SOD levels, while oLAb was not significantly different. Almost all plasma levels of oxidative stress markers, inflammatory proteins, and vascular endothelial growth factor (VEGF) were increased at chronic renal failure stages 3 to 5, indicating an early appearance of oxidative stress during the progression of renal insufficiency [79]. A decreased oLAb titer was observed in children on hemodialysis. Especially those patients with very low oLAb-titers (<137 mU/mL) indicated an increased hs-CRP level, and lower levels of albumin, apolipoprotein A-1, and high-density lipoprotein. The situation was even worse in patients with dyslipidemia [21]. Differentiation in hemodialysis patients with or without vascular calcification (VC) in the legs could be detected neither by oLAb (416.9 ± 287.1 mU/mL − VC vs. 341.5 ± 307.3 mU/mL + VC, *p* = 0.131) nor by the numerous biomarkers tested, apart from a decreased concentration of HDL. However, an inverse correlation between oxLDL and oLAb was found in this group of hemodialysis patients [80]. The oLAb titers in kidney graft recipients are significantly lower compared to controls. In the post-transplant period, these titers decreased further, most likely due to permanent immune-suppressive treatment, within the first six months and remained at this diminished level until the observation period of 24 months. Low pre-transplant oLAb titers (207 [89–855 U/L]) were associated with early graft loss on account of acute rejection versus patients with graft survival longer than three months (546 [79–16,748 U/L]) in addition to a higher frequency of ischemic heart disease (*p* < 0.05) and an increased chronic graft damage score of greater than 6. The authors highlight the protective effect of oLAb, which binds oxidatively modified lipids and thus protects the vessel walls from damage, as they were able to substantiate that a low oLAb titer is associated with an increased risk for complications in kidney graft recipients [81]. Another study with 71 post-renal transplant patients confirmed an identical oLAb titer (225 mU/mL; range: 40–850 mU/mL), which was significantly lower compared to that of hemodialysis patients (*n* = 33; 462 mU/mL; range: 89–850 mU/mL, *p* < 0.01) but higher than that of the control group (*n* = 89; 175 mU/mL; range: 45–350 mU/mL, *p* < 0.05), with the restriction that the oLAb titers of the control group were below the normal range of 200–600 mU/mL [75].

## 5. Cancer and oLAb

There was no association between oLAb levels and the risk of colon cancer, while elevated levels of oxLDL were associated with an increased risk of colon cancer, independent of confounding factors [82]. In colon cancer patients, an association with advanced age and increased oLAb titers, especially at the beginning of the disease at stages 1 and 2, depending on the localization, were reported. In contrast, oLAb titers were significantly lowered in advanced stages, i.e., in severe cases. The authors described this effect as an individual difference in the host’s immune response to cancer [10]. Increased oxidative stress was also demonstrated in patients with hepatocellular carcinoma, whose oLAb titers were bisected compared to those of the control group [83].

## 6. SIRS and Sepsis with Respect to oLAb

In intensive care unit patients, there was a significant increase in oLAb titers accompanied by decreasing inflammation parameters in contrast to non-survivors, which indicated an inverse sequence of events [84].

## 7. Thyroid and oLAb

Hypothyroidism is associated with an increased production of antibodies against oxLDL, compared to a healthy control group. On the other hand, a decreased oLAb titer was observed in hyperthyroid patients. Thus, thyroid dysfunction leads to oxidative stress, lipid peroxidation, and atherosclerosis, which was indicated by a decrease in TAC and an increase in total peroxides. Therefore, especially in hypothyroidism, the “response to injury” stimulates the immune system [85].

## 8. Multisystem Disorder—Behçet’s Disease with Respect to oLAb

Patients suffering from Behcet’s disease, a multisystem disorder with relapsing inflammatory processes and its predominant histopathology of vasculitis, are prone to oxidative stress. ROS overstrain the antioxidant system with a significant decrease in antioxidant enzyme activities such as catalase and superoxide dismutase, as well as glutathione, total antioxidant status, and shortened lag-time. As a counteraction to elevated lipid hydroperoxides, the oLAb-titer increases as an immunological stress response [86,87].

## 9. Congenital Disorder—Cystic Fibrosis with Respect to oLAb

In cystic fibrosis patients, oxidative stress was indicated through a decrease in retinol, α-tocopherol, and β-carotene, and a significantly increased and exorbitant oLAb, as well as TBARS. The authors concluded that an increased antibody titer against oxidized LDL might be an indication of an imbalance in the oxidant–antioxidant system, with an excess of oxidative stress in the presence of chronic lung inflammation in CF [88].

## 10. Chronically Inflammatory Diseases and oLAb

### 10.1. Morbus Crohn

In Crohn’s disease patients, oLAb titers better reflected the disease severity than oxLDL, and are positively correlated with the proinflammatory interleukin 1 (IL-1) (*p* = 0.038) and disease activity index (*p* = 0.035) [89].

**Table 1:** Antibodies against oxidized LDL (oLAb) from infancy to old age, reference values, ethnicity, sport, human pathologies, and effects of antioxidants—study results.

Author/Year	Study	Age	*n*	Subjects	oLAb (mU/mL)	Signif. (*p*)
Huang et al., 2013 [8]	AMI		38	Non-Restenosis	57.5 ± 12.5	
				Before (BA)	335 ± 257	
				Day 3	277 ± 185	
				Day 7	352 ± 279	
			18	Restenosis	57.4 ± 10.4	
				Before	181 ± 114	0.003
				Day 3	155 ± 92	0.001
				Day 7	177 ± 110	0.001
Diakowska et al., 2015 [10]	Cancer	57 (55–62)	35	Control	282 (219–409)	
			73	Total CRC	488 (286–1046)	
			52	Colon	578 (285–1131)	0.001 ^C^
						0.005 ^Rec^
			21	Rectum	352 (285–450)	0.005 ^Col^
				Prim Tumor:		
			32	Right Colon	774 (497–1148)	0.023 ^Rec^
		69 (67–76)	21	Rectum	353 (286–451)	
				T (progression):		
			11	T1 + 2	1133 (212–1203)	0.001 ^T4^
			21	T4	450 (349–687)	
Fialova et al., 2002 [19]	Pregnancy	27 ± 4.1	40	Control 1st TM	561.5 ± 424	
				Control 2nd TM	581.0 ± 434	
			26	Hypertension	348 ± 388	0.01
			49	Normotensive	579 ± 400	
Kara et al., 2008 [20]	T1-DM	6.6–18.1	20	Control	110 (37–235)	
		6.6–18	36	T1-DM	278 (37–1289)	0.001 ^C^
				Good Control	488 (51–1289)	0.001 ^C^
				Poor Control	183 (37–1207)	0.02 ^GC^
Cengiz et al., 2009 [21]	Hemodialysis	14.3 ± 2.7	20	Control	653 ± 721	
		15.1 ± 2.5	28	Patients	253 ± 306	0.014
				Norm Lipids	540 ± 397	
				Dyslipidemia	109 ± 57	0.001 ^NL^
Pincemail et al., 2000 [25]	Sport	21–64	123	Normal range	468 ± 318	
		24.6 ± 4.3	14	Athl Norm oLAb	321 ± 100	
		29.8 ± 5.7	16	Athl High oLAb	2829 ± 1916	0.0001
Steinerova et al., 1999 [27]	Neonates	20–29	20	Neonates	421 ± 172	
		20–29	20	Mothers (M)	340 ± 268	
Steinerova et al., 2001 [28]	Feeding			M (at birth):		
			6	Breast fed	803 ± 375	
			8	Formula fed	1418 ± 1271	
				Newborns:		
			6	Breast fed	657 ± 316	
			8	Formula fed	739 ± 605	
				M (3 Months):		
				Breast fed	721 ± 297	
				Formula fed	1025 ± 818	
				Newborns (3 M):		
				Breast fed	84 ± 36	
				Formula fed	4066 ± 2342	
Zima et al., 1998 [31]	Alcoholics	40 ± 8	60	Control	500 ± 52	
		42 ± 9	35	Alcoholics	406 ± 52	
Pincemail et al., 2007 [32]	Contraception	44.6 ± 2.5	119	Control	376 ± 383	
		44.4 ± 2.7	49	Oral contracept	442 ± 425	n.s.
		44.0 ± 2.1	41	Intrauterine dev	431 ± 388	n.s.
Miller et al., 2009 [33]	Ethnicity		259	Whites	377 (330–432)	
			169	African	526 (445–621)	0.05
			196	S-Asian	578 (495–675)	0.001
				Smokers	384 (316–468)	0.031
				Non-smokers	430 (471–596)	
Suzuki et al., 2003 [37]	Smokers	61.2 ± 10.2	158	Non-smokers	291 (194–452)	
			41	Smokers	177 (103–343)	n.s.
Yasunobu et al., 2001 [38]	Stenosis			Control	273 ± 21.4	
				Spasm	439 ± 45.3	
				Stenosis (St)	419 ± 48.7	0.05
				St non-smokers	275.6 ± 33.5	
				St smokers	472.2 ± 65.9	0.0449
				Stable angina	292 ± 33.5	
				Unstable angina	591 ± 97.9	0.005
Karolkiewicz et al., 2006 [39]	Overweight-	62–74	83	Young-old	363.81 ± 275.87	
	old males	75–83	26	Old-old	471.45 ± 274.07	0.05
Wonisch et al., 2012 [41]	BMI	33 ± 4	106	BMI < 19 (I)	613 ± 260	
		40 ± 10	1110	BMI 19–24.9 (II)	519 ± 81	
		46 ± 8	646	BMI 25–29.9 (III)	490 ± 69	0.05 ^(I)^
		49 ± 5	242	BMI 30–34.9 (IV)	453 ± 105	0.05 ^(I & II)^
		49 ± 10	84	BMI > 35 (V)	507 ± 305	
		41 ± 12	1283	Females	513 ± 369	
		43 ± 13	907	Males	485 ± 363	
Nansseu et al., 2017 [44]	Soccer	20.6 ± 3.1	18	March	653 (468–838)	0.006
				May	777 (553–1150)	
				July	1037 (901–1481)	
Schippinger et al., 2009 [45]	Ski Racers	26.1 ± 2.7	8	July	1036 ± 328	
				November	884 ± 552	
				December	439 ± 150	0.05
				January	856 ± 258	
Castaner et al., 2011 [53]	Olive oil	20–60	200	oxLDL↑/oLAb↓		0.001
				OOPC↑/oLAb↑		0.014
				PC↑/oLAb↑		0.023
Sohrab et al., 2017 [55]	Pomegranate	40–65	30	Control Baseline	132.2 ± 109.9	
				Control (6 W)	141.6 ± 115.3	
				T2-DM (BL)	125.3 ± 93.1	
				T2-DM (6 W)	97.4 ± 82.1	0.02 ^BL^
						0.05 ^C^
Resch et al., 2006 [57]	Rosuvastatin			Baseline	565.4 ± 511.8	
		57.8 ± 7.8	58	Ro (10 mg/12 w)	462.1 ± 392.8	n.s.
		58.2 ± 7.5	51	Ro (40 mg/24 w)	490.3 ± 380.0	n.s.
				Male (BL)	533.6 ± 562.8	
				Male (12 w)	466.4 ± 471.8	
				Male (24 w)	442.7 ± 442.6	0.05
				Female (BL)	595.4 ± 363.1	
				Female (12 w)	517.2 ± 352.8	
				Female (24 w)	593.7 ± 364.3	
Simonini et al., 1999 [59]	Sclerosis	51 ± 2.1	21	Control	89.3 ± 29.1	
		53 ± 3.8	63	SSc	309 ± 367	0.0001
			40	LSSc	351 ± 351	0.0001
			23	DSSc	207 ± 316	0.05
			30	DSSc early	428 ± 417	0.001
			33	DSSc adv	302 ± 89	0.0001
Andican et al., 2008 [60]	Atherosclerosis	47.67 ± 13.61	21	Control	266 ± 113	
		63.05 ± 9.13	21	PAD	357 ± 177	0.05
			11	Mild	316 ± 119	0.05
			10	Severe	403 ± 223	0.05
Trinker et al., 2010 [61]	PAD	67 ± 8	17	Fontaine IIb	355 ± 250	
		73 ± 8	10	Fontaine IV	341 ± 209	
			18	PTA successful	251 ± 190	0.05
Pawlak et al., 2012 [63]	Dialysis		29	Control	326 (111–1595)	
			16	CKD-CVD(+)	116 (43–218)	0.001 ^C^
			55	PD-CVD(−)	269 (116–1650)	0.01 ^CKD^
				PD-CVD(+)	170 (69–917)	0.01 ^C^
						0.05 ^CVD-^
						0.05 ^CKD^
			60	HD-CVD(−)	286 (77–2020)	0.01 ^CKD^
				HD-CVD(+)	197 (38–1702)	0.01 ^C^,
						0.05 ^CVD-^
						0.05 ^CKD^
Weinbrenner et al., 2003 [64]	CHD	23–92	32	Control	796 ± 1034	
		42–82	32	CHD	341 ± 350	0.05
Chen et al., 2008 [65]	IMT	52 ± 15	52	Low oLAb	190 ± 54	0.042
			83	Moderate oLAb	339 ± 55	
			65	High oLAb	1218 ± 147	
Shoji et al., 2003 [66]	Renal Disease		103	Endstage RD	387 ± 273	0.042
Bilgen et al., 2005 [68]	CAD	53.48 ± 10.22	31	Control	181 ± 91	
		57.6 ± 11.25	136	CAD (nvd)	332 ± 159	0.001
				CAD (svd)	242 ± 112	
				CAD (dvd)	253 ± 126	
				CAD (tvd)	222 ± 131	
Gomez et al., 2014 [70]	MI	61.9 ± 10.7	45	Control	447 (194–1081)	
		66.1 ± 7.6	34	MI patients	128 (103–235)	0.001
Gruzdeva et al., 2014 [72]	ST-MI		33	Control	206 (189–225)	
		50 (49–51)	135	A (1st day T1)	284 (251–312)	0.05
				A (12th day T2)	395 (374–423)	0.05
		58 (44–73)	115	B (T1)	334 (316–354)	0.05
				B (T2)	382 (369–399)	0.05
				C (T1)	484 (459–509)	0.05
				C (T2)	545 (521–574)	0.05
Cherubini et al., 1997 [74]	Stroke	76 ± 9	16	Control	505 ± 103	
		77 ± 8	12	Stroke Pat	246 ± 38	0.05
				Hypert Con	640 ± 163	
				Hypert Pat	199 ± 41	0.05
Klimak et al., 2011 [75]	Kidney TP		89	Control	175 (45–350)	
			33	Hemodialysis	462 (89–850)	0.01
			71	Post-Transpl	225 (40–850)	0.05
Pawlak et al., 2006 [76]	Hemodialysis		20	Control	210 (100–825)	
		60 ± 15	24	HD (total)	405 (130–1670)	
		63 ± 13	15	HD + CVD	420 (200–1670)	0.05
		58 ± 16	9	HD − CVD	380 (130–1340)	
Zezina et al., 2002 [81]	Kidney Graft		90	Recipients	766 (133–10,170)	
		45 ± 15	92	Pre-Transpl	539 (79–16,748)	0.05
				Post-Transpl	488 (37–15,680)	0.05
				Low-oLAb	<255	
Örem et al., 1999 [86]	Behcet’s	30 (17–50)	30	Control	187 ± 132	
		31 (16–50)	37	Patients	425 ± 365	0.05
Benabdeslam et al., 1999 [88]	Cyst. Fibr.	14 ± 6	40	Control	825 ± 1009	
		11.8 ± 7.2	76	Patients	8498 ± 14,347	0.005
Kural et al., 2003 [90]	Psoriasis	36.2 (24–48)	35	Control	225 ± 120	
		34.8 (27–43)	35	Patients	388 ± 176	0.002
Örem et al., 1999 [91]	Psoriasis	29 (15–58)	30	Control	162 ± 77	
		29 (15–58)	33	Patients	437 ± 345	0.001

Abbreviations: *n* (number of cases), oLAb (antibody oxidized low-density lipoprotein), AMI (acute myocardial infarction), Before (BA) (before primary angioplasty), CRC (colorectal cancer), T1-DM (type 1 diabetes mellitus), Athl Norm oLAb (athletes with normal oLAb), Athl High oLAb (athletes with high oLAb), Intrauterine dev. (intrauterine device user), BMI (body mass index), OOPC (olive oil phenolic compound), PC (phenolic compound), SSc (systemic sclerosis), LSSc (limited subset), DSSc (diffuse subset), PAD (peripheral arterial disease), CKD (chronic kidney disease), CVD (cardiovascular disease), PD (peritoneal dialysis), CHD (coronary heart disease), IMT (Intima Media Thickness), CAD (coronary artery disease), nvd (no vessel disease), svd (single vessel disease), dvd (double vessel disease), tvd (triple vessel disease), MI (myocardial infarction), ST-MI (ST-elevation myocardial infarction), TP (transplantation), HD (hemodialysis), Behcet’s (Behcet’s disease), Cyst. Fibr. (Cystic fibrosis).

### 10.2. Psoriasis

In psoriasis patients who suffer from a chronic and recurrent inflammatory skin disease, there is an increased atherogenic tendency due to a disturbance of the oxidant–antioxidant balance through a decrease in endogenous antioxidant enzymes, including the total antioxidant status in contrast to oxidative stress markers, e.g., malondialdehyde and lipid hydroperoxides were increased, as well as the antibody titer against oxidized LDL [90]. In this chronic inflammatory skin disease, which is associated with, amongst other things, dyslipoproteinemia and, in further consequence, with an increased incidence of cardiovascular disease, the oLAb titer was significantly increased in patients compared to healthy controls. The authors concluded that an imbalance in the oxidant–antioxidant system was responsible for an increased inflammation even though LDL-C levels of patients and controls did not differ significantly. The oLAb titer correlated significantly with the Psoriatic Area and Severity Index (PASI) score, indicating the clinical severity of the disease and concomitant inflammation due to elevated PMN elastase and alpha-1 antitrypsin levels [91].

### 10.3. Vaginitis

In 45 patients with recurrent thrush vaginitis, increased total peroxides (399 µmol) and oLAb titers (648 mU/mL) were reported with a corresponding drop in TAC. Through an antioxidant therapy, 77% of the patients became symptom- or recurrence-free [92].

## 11. Allograft Transplantation and oLAb

In liver and heart allograft transplantation patients, an inverse correlation was observed between malondialdehyde and oLAb (r = 0.61, *p* < 0.001 in the case of liver and *p* = 0.54, *p* < 0.05 in heart transplantations), indicating oLAb as a second barrier against lipid peroxidation [93].

## 12. Post-Traumatic Changes and oLAb

The lowest oLAb titers in patients with bone fractures and/or traumatic brain injury (TBI) were found in the first week. However, those with TBI had significantly higher titers than those with isolated fractures. Over four weeks, there was a significant gradual increase in all patients. However, an increase in the oLAb titer compared to controls was only significant in TBI patients in the third and fourth weeks [94].

## 13. COVID-19 and oLAb

After the infection with SARS-CoV-2, the oLAb titer significantly decreased in recovered and diseased COVID-19 patients, i.e., with an enhanced decrease in non-survivors, albeit not significantly different from survivors [95]. It should be mentioned that there was a persistent inverse correlation between the concentration of 4-hydroxynonenal and oLAb in the blood of non-survivors, compared to a positive correlation in survivors at the end of the observation period [96]. This effect was assigned to inflammation and the attack of free radicals, which were associated with alterations in lipid metabolism, thus resulting in systemic oxidative and vascular stress, and an exhaustion of the immune system. The authors concluded that oLAb titers may have a prognostic value in COVID-19 patients.

## 14. Conclusions

Over three decades, numerous clinical studies have determined the antibodies against oxidized LDL and established correlations with clinical symptoms and diverse other biomarkers. The oLAb titer in the general population is heterogeneous, while the titers within an individual are relatively constant. In this respect, the measurement of the oLAb titer is solicited as a robust biomarker, e.g., in annual health examinations. These physiologic autoantibodies serve to dispose of oxidatively modified molecules and cells, whereby binding to the antigen is accompanied by a reduction in the titer. This immunological support is a second barrier and is particularly useful in cases of increased oxidative stress, e.g., in the case of intensive care patients or myocardial infarction patients. The oLAb is convenient for monitoring the course of these severe illnesses to monitor progress in recovery. Monitoring these antibodies is further indicated in heavy workers and elite sports, where athletes are exposed to increased oxidative stress due to heavy physical exhaustion, which is also associated with performance. Future research should also focus on antioxidants and their correlation to oLAb, especially as there is clear evidence that antioxidant supplementation can significantly influence antibody titers. In this regard, it should be noted that oxidative stress takes place at different levels, and, therefore, additional OS biomarkers and the knowledge of the antioxidant capacity are necessary for a profound assessment. However, antibody research is non-exclusive beneficial in diagnostics but could also significantly contribute therapeutically to the fight against oxidative modifications in the future through monoclonal antibodies.

## Data Availability

Not applicable.
